# Supplementary material for: Development of an orally-administrable tumor vasculature-targeting therapeutic using annexin A1-binding D-peptides
Source: PLoS One. 2021 Jan 6;16(1):e0241157. doi: 10.1371/journal.pone.0241157 (PMC7787448; doi:10.1371/journal.pone.0241157)
Supplement: S3 File — (PDF) [file pone.0241157.s003.pdf]

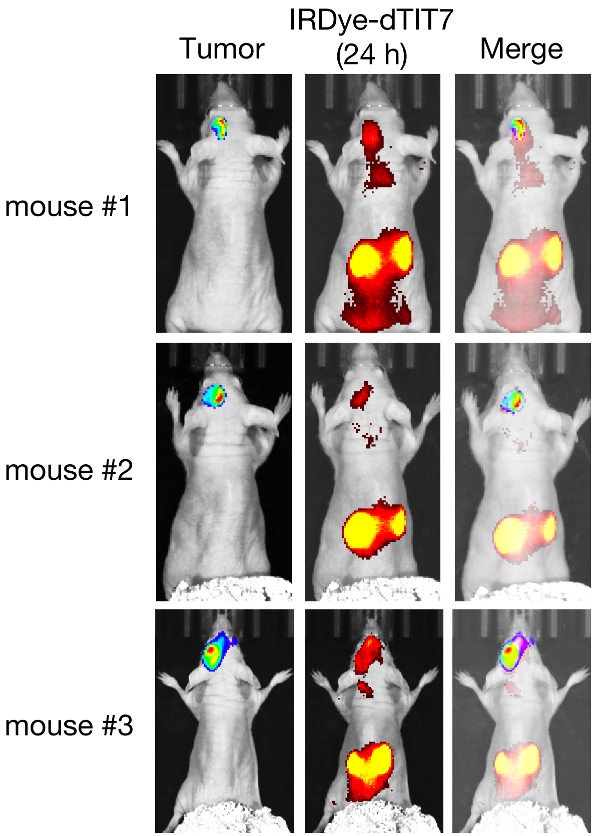

Whole body imaging of brain tumor-bearing mice by IRDye-dTIT7 conjugate. C6-Luc cells were injected into the brain of nude mouse. Upon 24 h post injection, IRDye-dTIT7 was injected intravenously.
