# Supplementary material for: Development of an orally-administrable tumor vasculature-targeting therapeutic using annexin A1-binding D-peptides
Source: PLoS One. 2021 Jan 6;16(1):e0241157. doi: 10.1371/journal.pone.0241157 (PMC7787448; doi:10.1371/journal.pone.0241157)
Supplement: Attachment — Submitted filename: Responces to the editor.pdf [file pone.0241157.s005.pdf]

Additional Editor Comments:

1. Is there a difference between Anxa1 and ANXA1? If not, please unify the format.
R: We have now unified the format to ANXA1.

2. Please add explanation about why dLRF7 was not further investigated into the discussion. It is worthwhile to elaborate the peptide selection criteria as well.
R: Our initial criteria for peptides selection had been (1) the number of phage clones selected by phage library screen (Fig 1D), (2) binding affinity to ANXA1 (Fig 2CD), and (3) in vivo tumor targeting activity (Fig. 4). We did not further investigate dLRF7 because it did not target tumors in vivo (Fig. 4B) and thus did not meet requirement #3. We now state this explicitly in the manuscript on lines 349-353.

3. Figure resolution is quite bad in the pdf file. Please ensure that they are of sufficient resolution in the final version.
R: We have now uploaded all tiff-formatted figures to the PACE program and converted them for optimal resolution.

4. Explanation of why IRDye-conjugated dTIT7 targeted the kidney should be in Discussion. It is also worthwhile to propose experiments on verify possible mechanism.
R: We have now added the following lines 438-443 to the Discussion. "In this study, we showed that IRDye-dTIT7 can be used for brain tumor detection by whole body imaging (Fig. 6). As unconjugated IRDye does not target normal mouse organs, the kidney targeting of IRDye-peptides may be caused by a unique structure created by IRDye/dTIT7 conjugates that binds to an unknown receptor expressed on the vasculature surface of normal kidney. This hypothesis is supported by our previous study showing that intravenously injected fluorescent Alexa488-IF7 targeted brain tumors but not kidney (9).

5. It is still not entirely clear why D-peptides were chosen over L-peptides for this study. Could you please further elaborate your reason?
R: Our previous study showed that IF7-SN38, which is the ANXA1-binding L-peptide IF7 conjugated with the anti-cancer drug SN38, is unstable in vivo (8). We hypothesized that replacing the IF7 with a D-peptide would improve the pharmacological activity of IF7-SN38 by increasing peptide stability. In fact, the retro-inverso form of IF7 (RIF7) reported by Chen et al. (12) was designed to address this concern. However, in their report, fluorescent-RIF7 showed significant non-specific organ targeting. Thus we undertook a different strategy employing D-peptides. We state these on lines 71-79.

6. Line 79, please provide more background info about retro-inverso. This is not readily understandable by researchers slightly outside the field.
R: The retro-inverso technique replaces L- amino acids by D-amino acids, while reversing the primary sequence of the peptide. We have added a sentence stating this and new reference reference #11 to the revision, line 75-76.

7. Regarding negative control of Fig 4, what is the localization of IRDye? Also, are there biological replicates? If so, please include them in the SI.
R: According to the manufacturer's information. IRDye localizes diffusely to entire mouse body. We mentioned this on lines 333-334. We include an additional figure of whole body imaging as Supporting Information 3.

8. Could you please provide a calculation on how the diversity of the phage library is calculated? Was each amino acid coded by NNN or NNK?
R: Theoretically, the diversity of the phage library is calculated as 20^n, where n is the number of peptide residues displayed on the phage surface. As we used a 7-mer peptide library, that diversity is 20^7=1.28^9. We now provide this equation in the Method section, line 112.

9. Lastly, the manuscript will benefit from language editing or thorough check of grammar and the use of language.
R: We have asked a professional scientific editor, Dr. Elise Lamar, to check grammar and English usage.
