# Supplementary material for: Development of an orally-administrable tumor vasculature-targeting therapeutic using annexin A1-binding D-peptides
Source: PLoS One. 2021 Jan 6;16(1):e0241157. doi: 10.1371/journal.pone.0241157 (PMC7787448; doi:10.1371/journal.pone.0241157)
Supplement: Attachment — Submitted filename: Response to reviewers.docx [file pone.0241157.s004.docx]

Response (R) to each criticism (C) raised by reviewers.

Reviewer #1: 
C: 52-53 There should be elaboration here into the nature of origin of the 7mer IF7 is at lays the foundation of this work. Was this discovered by screening? Is it derived from natural binding protein?

R: The sequence of IF7 or IFLLWQR was identified by peptide display phage library screening during a study of carbohydrate-mimetic peptides for selectins (carbohydrate-binding proteins). Initially, we thought that the peptide receptor on the endothelial surface is e-selectin. Further experiments revealed that the receptors were instead pre-mRNA splicing factors and Anxa1. This information is now provided in the Introduction (lines 56-62) of the revised manuscript. Also, new references #4-6 have been added relevant to these issues.

C: 58-59 This statement should be explained by the relation of transcytosis to the blood brain barrier – otherwise this lacks context for the non-expert.

R: We have now revised this text (lines 68-70) to read: “These observations led us to hypothesize that an IF7-conjugated drug would overcome the blood-brain barrier (BBB) by transcytosis to eradicate brain tumors.”

C: 70 Literature indicates that residues 2-26 of ANXA1 of the N-terminal domain serves as the second membrane binding site, with accompanying solution NMR and CD to show alpha helicity. It is unclear why residues 1-15 were chosen specifically.

R: Relevant to this question, we have added the following sentences with new references (#11, 12) to the revision at lines 84-87: “A synthetic Ac2-26 peptide from the Anxa1 N-terminal domain reportedly inhibits recruitment of neutrophils in several models of experimental inflammation [15]. As Lim *et al*., reported that Ac2-12 mimics the activity of Ac2-26 [16], we tested a series of synthetic Anxa1 N-terminal peptides and found that the N-terminal 1-15 residues are sufficient for IF7 binding [9].

C: 223-227 TIT7 sequence was the focal point for most of the experiments due to the majority enrichment in the phage clones Fig1D. dTIT7 binds to ANXA1 with a Kd of 46.4 nM. Fig 2D shows dLRF7 has tighter binding at 31.2 nM. It is unclear why dTIT7 was taken forward at this point and dLRF7 was disregarded. Plus, error analysis is needed.

R: It is true that dLRF7 showed stronger binding than dTIT7 based on a QCM assay. However, we did not switch our focus from dTIT7 to dLRF7 based solely on the QCM result, as dTIT7 was the most enriched in the phage library screening (Fig. 1D), and whole-body imaging (Fig. 4) indicated that dTIT7, but not dLRF7, targeted brain tumors. Thus, no revision was made.

C: 270 Since the activity of the D-peptide drug conjugates is significantly less than the original L-IF7 conjugates – the utility of the D-peptide vehicles presented in this work could be demonstrated with pharmacokinetic analysis.
Dissociation constants of Fig 2A and C could be larger as it is difficult to read.

R: We appreciate the reviewer’s comment. We are eager to address pharmacokinetics of dTIT7 in future studies. We state this briefly in the revision (at line 443).

Also, we have now enlarged font size in Fig. 2A and C.

C: 229 NMR at 500 MHz frequency does not provide sufficient resolution to make a strong argument of solution-phase binding. The differences between the spectra are not clear. Also, there is no evidence that MC16 adopts an alpha helix in solution. Ideally, the work would include CD spectra of MC16 and D-MC16 under similar conditions to literature NMR (50% TFE/Water or 10mM SDS) and similar conditions used for any analysis of solution-state binding. Otherwise, any indicated binding between dTIT7 and the MC16 can be argued as non-specific interaction.

R: To strengthen our argument relevant to solution phase binding, we have added the following two sentences to the revision. “This indicates a significant peak shift, possibly due to a direct ordered interaction and/or to a specific conformational change” (287-289). “Overall, these results indicate that dTIT7 and MC16 associate in solution, and do not undergo a merely disordered attraction.” (293-294)

The reviewer suggested that we obtain NMR data with 50% TFE/Water or 10mM SDS. However, we chose not to do this as these conditions are not physiological. Thus, no revision was made on this issue.

C: 246-257 D-TIT7 accumulates in the kidney vasculature at a significantly higher level than the brain tumour. Is this as indictment of using Annexin A1 as a surface marker in malignant tumour vasculatures. Or is this a question of off target effects of the peptide-conjugates. An explanation is not offered in the discussion.
R: Currently, we do not have data to explain why IRDye-conjugated dTIT7 targeted the kidney. We state this in lines 331-332 in the revision.

C: Overall, the work is original and demonstrates a clear application of utilising short protein domains to identify D-peptide binders for drug delivery leads – albeit with some concerns as to whether MC16 is a good enough model of the N-terminal binding domain in this case. The points made on the introduction should be addressed prior to publication, as it impacts the communication of the presented work.

R: We greatly appreciate you comments.

C: Lastly, in the introduction, the authors need to explain why D-peptides, rather than cyclic L-peptides which have also been suggested to be biologically stable, are used.

R: We briefly discussed use of cyclic L-peptide in Introduction and now provide a rationale for choosing to employ D-peptides (see lines 77-79 in the revision).

I think this manuscript is suitable for publication in PLoS one.

Reviewer #2: The paper by Nonaka et al., on “Development of an orally administrable tumor vasculature targeting therapeutic using annexin A1 binding D-peptides” deals with an important step in tumor theranostic and this is through orally administrable drug/peptide. It is well thought study with profound inside however there are some missing links. Hence, I recommend it to publish with minor revision considering the impact and importance of the outcome.

Minor comments:
C: (1) What is the genesis of selecting ANXA1-binding D-type peptide apart from getting this from Phage library? Is there availability of D-type peptides in tumor related cases; it should be clearly brought in in “introduction”?

R: Apart from phage library screen, the retro-inverso strategy was applied to generate D-type Anxa1-binding peptide by Chen et al. (cited as reference #11). We described this strategy in the Introduction (lines 79-80, in the revision).

C: (2) Why linear 7-mer peptides library were chosen? Can one use shorter or longer length? What if peptides are cyclic?

R: We chose a linear 7-mer peptide library as we wanted to select carbohydrate mimetic peptides. Most carbohydrate antigen epitopes consist of 3-4 carbohydrate residues, a size comparable to 7 amino acid residues. We did not use a phage library larger than 7-mer peptides, as the volume of the library becomes exponentially larger. We now cite our earlier publications relevant to carbohydrate mimetic peptides in the Introduction of the revision, line xx-xx, (ref #5).

C: (3) In Figures 3 as well as in results section, authors mention about IF7 binding to Anxa1 dimer. What is stoichiometry of binding and does binding break the dimerization or leads to multi-merization?

R: Anxa1 dimerization was shown by isothermal calorimetry analysis in our previous publication (Supplemental Fig. S3 in ref #7). The stoichiometry of IF7 to ANXA1 was 0.514: 1.00. IF7 binding led to ANXA1 dimerization. We now state this in the legend to Fig. 3 (308-309).

C: (4) From Figure 1D, there are ~53% other peptides? Are they just random or have homology to the prominent ones; should not be they considered for study?

R: All peptide sequences are shown in Supplemental Information 2. Many are short and overlap with dTIT7. These are noted in the legend to Fig. 1D (line (??) 256 in the revision).

Reviewer #3: In this manuscript, Nonaka et al. developed an orally-administrable D-peptide-drug conjugate targeting ANXA1 receptor expressed on cancer cells. They first identified peptide sequences that bind to a D-peptide derived from N-terminal domain of ANXA1 through mirror-image phage display. Interaction between D-peptides and L-ANXA1 peptide and protein was then confirmed by ELISA, QCM and NMR. The authors tested the localization of selected D-peptides in mouse whole body using fluorescence imaging and confirmed targeted localization. Although intravenous injection of D-peptide-drug conjugate was not effective probably due to an insufficient dose, they finally observed therapeutic effect of GA-dTIT7 through oral administration.

The results shown in this manuscript is interesting, convincing, and suitable for the readers of PLOS one. Therefore, this report would be acceptable after the authors addressed specific comments and questions shown below.

C: 1) In Figure 4, there are no control experiments using some D-peptide with random sequence. To clarify the effectiveness of selected D-peptide for targeted localization, the authors should add a result of the control experiment, if possible.

R: In our previous study of IF7, we used reverse IF7 or RQ7 peptide as the negative control of IF7. However, RQ7 did not serve true negative control when it was conjugated with Alexa 488. We therefore used C(RR) peptide as the negative control in the subsequent study (ref xx). By a similar reason, we had difficulty in designing the negative control peptide for dTIT7, and thus did not perform an experiment with a control peptide in this report. No revision is made on this issue.

C: 2) In the caption of Figure 6, “B.” should be bold. “C. B16-Luc cells,,,” and “D. C6-Luc cells,,,” should be “D. B16-Luc cells,,,” and “E. C6-Luc cells,,,”, respectively.

R: Thank you for detecting these errors. They have now been corrected.

C: 3) How much is the diversity of phage library used in this study? The authors should clarify it in the text.

R: The diversity of the library is 1.28x10^9. This is now stated in the revised Methods section at line 111.
